# Region-Specific Disruption of Adenylate Cyclase Type 1 Gene Differentially Affects Somatosensorimotor Behaviors in Mice

**Published:** 2014-12-30

**Authors:** Hiroyuki Arakawa, Fatih Akkentli, Reha S. Erzurumlu

**Affiliations:** Department of Anatomy and Neurobiology, University of Maryland School of Medicine, Baltimore, Maryland 21201

**Keywords:** AC1, barrels, knockout mice, somatosensory cortex, thalamus, whiskers

## Abstract

Adenylate cyclase type I (AC1) is primarily, and, abundantly, expressed in the brain. Intracellular calcium/ calmodulin increases regulate AC1 in an activity-dependent manner. Upon stimulation, AC1 produces cAMP and it is involved in the patterning and the refinement of neural circuits. In mice, spontaneous mutations or targeted deletion of the *Adcy1* gene, which encodes AC1, resulted in neuronal pattern formation defects. Neural modules in the primary somatosensory (SI) cortex, the barrels, which represent the topographic distribution of the whiskers on the snout, failed to form (Welker et al., 1996; Abdel-Majid et al., 1998). Cortex- or thalamus-specific *Adcy1* deletions led to different cortical pattern phenotypes, with thalamus-specific disruption phenotype being more severe (Iwasato et al., 2008; Suzuki et al., 2013). Despite the absence of barrels in the “barrelless”/*Adcy1* null mice, thalamocortical terminal bouton density and activation of cortical zones following whisker stimulation were roughly topographic (Abdel-Majid et al., 1998; Gheorghita et al., 2006). To what extent does patterning of the cortical somatosensory body map play a role in sensorimotor behaviors? In this study, we tested mice with global, cortical, or thalamic loss of AC1 function in a battery of sensorimotor and social behavior tests and compared them to mice with all of the whiskers clipped. Contrary to intuitive expectations that any region-specific or global disruption of the AC1 function would lead to similar behavioral phenotypes, we found significant differences in the degree of impairment between these strains.

## Introduction

Cyclic AMP signaling plays an important role in the patterning of somatosensory maps in the brain. Genetic loss of function studies in mice have shown that neuron-specific adenylate cyclase (AC) 1 is necessary for the formation of whisker- and paw-related neural modules (the barrels) in the face and body representation areas of the primary somatosensory (SI) cortex (Welker et al., 1996; Abdel-Majid et al., 1998).

The mouse SI cortex is characterized by a patterned, barrel-shaped distribution of layer IV neurons, which surround patchy aggregates of thalamocortical axon (TCA) terminals. TCA terminal arbors synapse with barrel cell dendrites that both orient to and embrace them. A spontaneous mutation, which occurred in ICR stock at Université de Lausanne (Switzerland), led to a “*barrelless*” mouse phenotype (Welker et al., 1996). A couple of years later, Abdel-Majid et al. (1998) identified disruption of the *Adcy1* gene in the *barrelless* mice. Later loss-of-function studies in the visual system revealed an important role for AC1 in retinotopic refinement (Ravary et al., 2003; Dhande et al., 2012). Further, cortex or sensory thalamus-specific conditional gene knockouts showed differential effects on barrel patterning and refinement (Iwasato et al., 2008; Suzuki et al., 2013). The authors who made and published the conditional knockout mice called them CxAC1KO (for cortex-specific *Adcy1* null) and ThAC1KO (for thalamus-specific *Adcy1* null). We herein use the same terminology to keep in line with these reports, and for consistency, we refer to the global knockout, *barrelless*, line as AC1KO.

**Figure 1:**
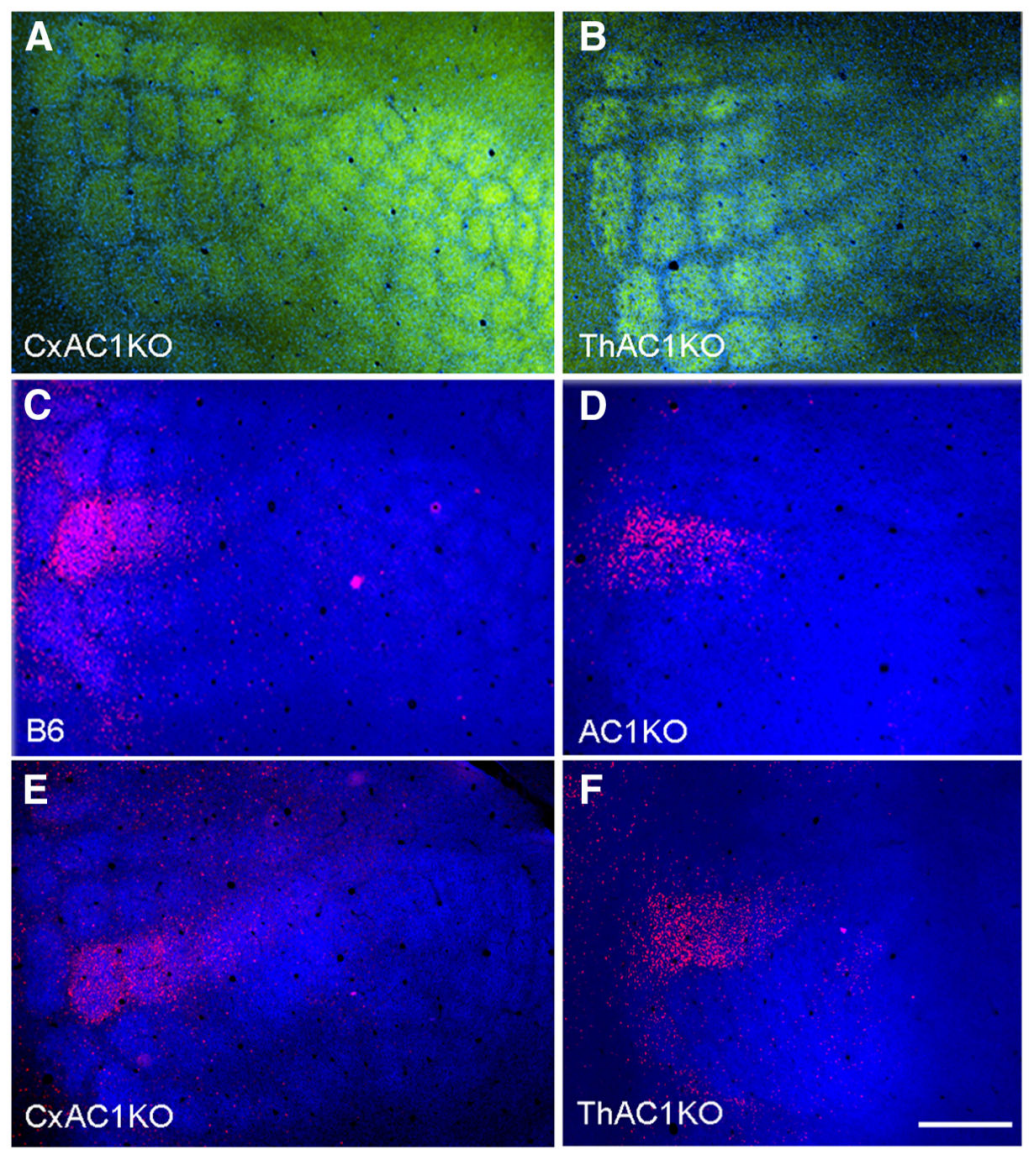
Barrel patterns and whisker-related activation in the barrel cortex. ***A***, ***B***, VGLUT-2 (green, for TCAs) and Neun (blue, for neurons) double immunostaining reveals that both the TCA terminal segregation and cellular organization into barrels appear normal in CxAC1KO cortex (***A***). In the ThAC1KO cortex, cellular patterning is absent (note the rather uniform distribution of the Neun-labeled blue cells) and there is some patterning of the TCA terminals (green; ***B***). ***C***, ***D***, VGLUT-2 (blue) and c-fos (pink) immunolabeling in the barrel cortex of a B6 (control, ***C***) and AC1KO (***D***) mice following row C whisker activation. Note that in the AC1KO cortex, a topographically appropriate zone is activated, although there is no VGLUT-2-related patterning. ***E***, ***F***, VGLUT-2 (blue) and c-fos (pink) immunolabeling in the barrel cortex of exemplary CxAC1KO and ThAC1KO mice following row C whisker activation. Note that the activity pattern in the CxAC1KO cortex is similar to B6 control, while that of the ThAC1KO cortex is similar to the AC1KO cortex. Scale bar, 200 *μ*m.

In CxAC1KO mice, layer IV neurons form grossly normal barrels, and TCAs fill the barrel hollows (Iwasato et al., 2008; see also Fig. 1). However, these authors noted impaired dendritic asymmetry of barrel neurons and deficits in postsynaptic maturation of the thalamocortical synapses (Iwasato et al., 2008). Morphologic analyses in the ThAC1KO mice revealed a barrel cortex phenotype similar to that of the *barrelless* mutant or AC1KO mice, namely, wider terminal arborization of TCA terminals and an absence of barrels as cellular structures (Suzuki et al., 2013).

Curiously, earlier studies in AC1KO mice indicated that TCA terminal arbors, while covering expansive cortical territory, had peaks of bouton density in topographically correct positions and a 2-deoxyglucose labeling study showed topographically aligned activation of the cortical region following whisker stimulation (Abdel-Majid et al., 1998; Gheorghita et al., 2006). If, in the most drastic barrel cortex phenotype, the global AC1KO, somatotopy of the TCA projections are maintained, what is the role of patterning that is absent in these mice? How does the loss of pattern in the somatotopic cortical map or how do differential pattern deficits following cortical or thalamic loss of AC1 function affect sensorimotor behaviors? We provide answers to these questions by comparing the performance of AC1KO, with normal (WT-C57/Bl6-B6) and whisker clipped (WC) B6 mice and those of Cx- and ThAC1 knockout mice (both in B6 background) with their control (AC1^flox/flox^) littermates. We ran these lines of mice in a variety of sensorimotor behavior, whisker-related behavior, motor performance, and social behavior tests and found differential levels of impairment in sensorimotor, whisker-related, and social behavior tasks, but not in general motor ability tasks.

## Materials and Methods

### Animals

Mice were housed in an AAALAC accredited laboratory facility. We obtained B6 mice from The Jackson Laboratory, and maintained them in our breeding colony. All mutant mice were obtained from RIKEN, Japan (generously provided by Drs. T. Iwasato and S. Itohara) and maintained in our breeding colony. AC1^−/−^ (AC1KO) mice were derived from AC1^Chr(177)^ (heterozygous) pairs (Iwasato et al., 2008); 5HTT^cre/+^AC1^flox/−^ (ThAC1KO) mice were derived from breeding 5HTT^cre/+^AC1^Chr(177)^ mice with AC1^flox/flox^ mice (Suzuki et al., 2013); and EMX^cre/+^AC1^flox/−^ (CxAC1KO) mice were derived from breeding EMX^cre/+^AC1^Chr(177)^ mice with AC1^flox/flox^ mice (Iwasato et al., 2008). Generation of the mutants and whisker-related pattern formation along their somatosensory systems has been published in detail (Iwasato et al., 2008; Suzuki et al., 2013). We determined the genotypes by PCR from tail lysate DNA samples, using the primers published by the investigators who generated the mice (Iwasato et al., 2008; Suzuki et al., 2013). All animal procedures were performed according to the regulation of the authors’ university’s animal care committee.

### Whisker stimulation-induced immediate early gene expression and immunohistochemistry for pattern formation

To confirm topographically aligned activation of the somatosensory cortex following whisker stimulation, we used a procedure similar to that used in rats (Staiger et al., 2000, 2002). Mice had all their whiskers clipped unilaterally except the caudal whiskers in C row. The next day, they were introduced to an “enriched environment,” namely a larger shoebox cage filled with numerous objects and tunnels and pipes. After an hour-long exploration period, mice were euthanized, perfused with aldehyde fixatives.

We flattened cortices between microscope slides and sectioned at 50 *μ*m thickness with a vibratome (Leica 1000S). After several rinses in phosphate buffer (PB), we incubated the free-floating sections in antibody solutions at 4 °C for 48 h. We performed triple immunostaining using antibodies against NeuN for neuronal labeling, vesicular glutamate transporter 2 (VGLT-2) for thalamocortical afferent terminals in the barrel cortex, and c-fos for activity-dependent immediate early gene expression. The primary antibodies were rabbit polyclonal c-fos antibody (Ab) (1:500; Santa Cruz Biotechnology), guinea pig polyclonal Ab VGLUT-2 (1:500; Millipore Bioscience), and mouse monoclonal Ab NeuN (1:500; Millipore Bioscience). After primary Abs were washed from the sections, we applied fluorescent secondary Abs (FITC-conjugated donkey anti-guinea pig, 1:80; Cy3-conjugated donkey anti-rabbit, 1:125; Alexa647-conjugated donkey anti-mouse, 1:125; all from The Jackson Laboratory) for 1.5 h. Afterward, we rinsed the sections in PB several times, mounted them onto glass slides, and covered with glass.

We examined the sections under epifluorescence and photographed regions of interest, using filters of different wavelengths appropriate for the fluorescent tag of each secondary antibody.

### Behavioral testing

Mice were group-housed (3–5 per cage) in standard cages (28 × 17 × 12.5 cm) with filter-top lids. All mice received water and standard rodent chow *ad libitum*. The housing room was environmentally controlled on a 12:12 h light: dark cycle (06:00 – 18:00 h lighting) at a temperature of 21 °C, relative humidity of 50–60%.

In behavioral experiments, we used all male mice aged 9 – 12 weeks. The subjects were assigned into two sets of group comparisons. In the first set, we used B6 mice with intact whiskers (WT; *n* = 8), B6 mice that had all their whiskers clipped 1 d before testing (WC; *n* = 9), and AC1KO mice with intact whiskers (*n* = 9). We performed this group comparison to explore whether intact whiskers play a role in behavioral tasks (WT vs WC) and to what extent the global AC1 loss of function (AC1KO) affects behavioral performance (WT vs AC1KO). The second group comparison was between conditional knockouts, CxAC1KO (*n* = 8), ThAC1KO (*n* = 8), and AC1^flox/−^ mice (*n* = 8) that were littermates of Th or CxAC1KO mice. This comparison was made to differentiate between the thalamus- or cortex-specific AC1 deficiency-related behavioral performances.

On each day of testing, mice were moved to the experimental room and left undisturbed for 30 min before testing. In line with general guidelines of behavioral phenotyping strategies in mutant mice (Crawley, 2008), we tested the mice in tandem with small groups. We ran three cohorts of mice at three different times. The investigator was blind to the genotypes of the mice throughout behavioral testing and analyses.

The behavioral test battery included the following: (1) general sensorimotor tests, such as gap crossing, edge approach, swimming, and sticky paper test; (2) whisker-specific sensory tests such as whisking patterns and object shape and texture discrimination; (3) general motor ability tests, such as wire hanging and open field exploration; and (4) social behavior test. The interval between tests for individual mice was set for at least for 1 h during the test battery. We describe the specific tests below.

### Gap-crossing test

The gap-crossing test (Hutson and Masterton, 1986) adapted to mice by Barnéoud et al. (1991) is a specific test for cortical whisker function. The test consists of a series of trials requiring the subject to cross variable distance gaps (Troncoso et al., 2004). We placed individual mice in the center of an elevated lane (4 cm diameter) connected to a safe platform. The gap distance between the lane and the platform was changed from 0 to 6 cm in trials by 1 cm increments. We measured the distance of the gaps that mice were able to cross to the safe platform. The trials were done under infrared lighting so that the mice used tactile, whisker-sensation cues to detect and measure the gap without any visual cues.

### Edge approach test

This test is principally based on the postural reflex and limb placement tests (Gerlai et al., 2000). Individual mice were picked up by the tail and slowly lowered toward the edge of a desk (1 cm/5 s, measured by a stopwatch and ruler). They were stopped just before any tactile stimuli could indicate the approaching surface. Then they were lowered further so that their whiskers could touch the surface. We repeated the procedure four times, videotaped their behavior, and measured the distance of the body position from the edge of the platform that they were able to reach.

### Swimming test

Swimming test for rodents is used in a variety of contexts, including motor coordination and whisker use to balance while floating in the water (Carvell and Simons, 1990; Bialy and Beck, 1993). We placed mice individually in a 2 L Pyrex glass beaker containing 1.8 L of water (maintained at 24 ± 1 °C) for 5 min. We videotaped their performance and later analyzed swimming, including paddling with forelimbs and hindlimbs and floating (no paddling).

### Sticky paper test

We used the sticky paper test to measure tactile responses to an adhesive tape stuck on the palmar surface of the hind paw (Komotar et al., 2007). We performed the test in the home cage. An adhesive-backed label (0.5 cm square) was placed on the palmar side of the hind paw. We recorded the latency of the first reaction to the stimulus (paw lifting, sniffing, biting, or removal).

### Whisking test

We placed a small, clear glass cup in the cage sideways and the mouse was allowed to investigate inside and around the cup. Next, we placed other small objects (head of a tooth brush and a plastic cup) in the cage and the mouse investigated them. We videotaped the whisking behavior of the freely moving mouse at 120 fps with a high-speed camera (EX-FH100, Casio), and later analyzed whisking patterns. The frequencies and the types of whisking toward the objects were counted.

### Object-shape discrimination test

We conducted this task for three trials in the home cage under red lighting. During the first trial, we placed two objects (e.g., plastic Easter egg, toothbrush) in opposite corners of the cage, and the mouse was allowed to explore the objects for 5 min. After an interval of 1 h, the mouse was run on the second trial, which now contained one of the familiar objects from the first trial in the same location and a new object (e.g., a small pot, glass cup, black-colored cap, or D-type battery) that replaced the second object from the first trial. On the third trial, after 1 h interval, one familiar object and a new object were presented. The order of the objects was counter-balanced for each animal.

### Texture-discrimination test

This test was done in the same way as the object-shape discrimination, except the lighting conditions and the objects were different. The objects placed in the home cages were plastic cups (3 cm diameter and 5 cm height) covered with different materials and textures. The mice explored different textures with their whiskers under infrared lighting condition. The material cover of the cup for the control condition in trial 1 was sponge, and the covers for unfamiliar surface textures on trials 2 and 3 were metal mesh, plastic tips, silicon-brush, terry cloth, and cardboard.

### Wire-hanging test

This task is used to evaluate grasping ability, forelimb strength, and physical abilities in body coordination (Crawley, 1999). The test started with placement of the mouse on a wire cage top. We then inverted the cage top above the home cage so that the mouse hung from the wires. We taped the hind paws of the animal so that the mouse used only its forelimbs to suspend its body hanging from the wire cage top. We recorded the mean time of suspension (or the latency to when the mouse fell to the cage floor) in three trials per session. A maximum cutoff latency of 60 s was recorded if the mouse did not drop to the cage floor. We took the average of the time in three trials as a representative value for each mouse.

### Open-field test

To assess exploratory locomotor activity in a novel place, we used the open-field test for each mouse. This test is used to determine gross locomotion, exploratory habits, and general activity levels following various experimental manipulations or drug treatments (Mothes et al., 1996; Saitoh et al., 2014). The open-field apparatus was an empty clear Plexiglas arena (28 × 24 × 15 (height) cm), illuminated by a small red lamp. The floor of the chamber was divided into equal size quadrants by lines. We placed individual mice in the center of the field and recorded the freely moving behavior for 5 min. We measured the number of line crossings. In this test, our aim was to observe exploratory behavior, not general activity levels. Whisker function is closely associated with exploratory tendency but not with general activity levels, thus we recorded the behaviors for a short duration.

### Social behavior test

We conducted this test following the open-field test. Mice were kept in the chamber after the open-field test and an “intruder,” stimulus male (B6 mouse unfamiliar with the subject) was introduced into the chamber. We videotaped the behaviors of the animals during a 10 min test session and later analyzed the video. We counted the duration of the contacts when the subject mouse approached the intruder. We also calculated the ratio of appearances for flight responses to the intruder’s approaches from the front and back of the body.

### Statistical analysis

All data are expressed as mean ± SEM one-way or two-way ANOVA was used for the statistical analysis (Table 1). We used the Bonferroni test for the *post hoc* comparisons when needed. For the comparison between AC1KO and WT in whisking test, *p* values were calculated with unpaired Student’s *t* test with equal variance. For all tests, differences were accepted as significant at *p* < 0.05. Experimental design was random-design.

## Results

The investigators who generated the AC1KO, CxAC1KO, and ThAC1KO mice have described the associated barrel cortex phenotypes (Welker et al., 1996; Abdel-Majid et al., 1998; Iwasato et al., 2008; Suzuki et al., 2013). In these reports, the most pronounced pattern defects were seen in AC1KO and ThAC1KO mice. Functional 2-deoxyglucose labeling and analyses of thalamocortical axon terminal bouton densities revealed a rough whisker-specific topography even in the absence of the barrels in the AC1KO mice (Abdel-Majid et al., 1998; Gheorghita et al., 2006). We confirmed these findings by immunohistochemical staining of flattened cortices through the barrel region and by examining c-fos activity following row C whisker activation. As illustrated in Figure 1, both TCA terminal and barrel patterns are present in the CxAC1KO mice, but only a partial TCA terminal patterning without barrel (cellular) patterning is seen in the ThAC1KO mice. In AC1KO mice, there is no cellular or TCA terminal patterning, but even so, use of row C whiskers alone induces c-fos expression in a band equivalent to the location of row C representation in the barrel cortex (Fig. 1D).

The main purpose of our study was to compare the behavioral phenotypes of different lines of AC1-deficient mice to determine if loss of AC1, in a globally or region-specific manner, had similar or differential effects. Mice use multiple sensory modalities to recognize the environment surrounding them, including visual, sound, odor, sensory motor, and tactile cues. We set up a test battery to compare the behavioral performances of these mouse lines in relation to wild-type and all whiskers-clipped mice.

### Whisker sensorimotor ability tasks

**Figure 2:**
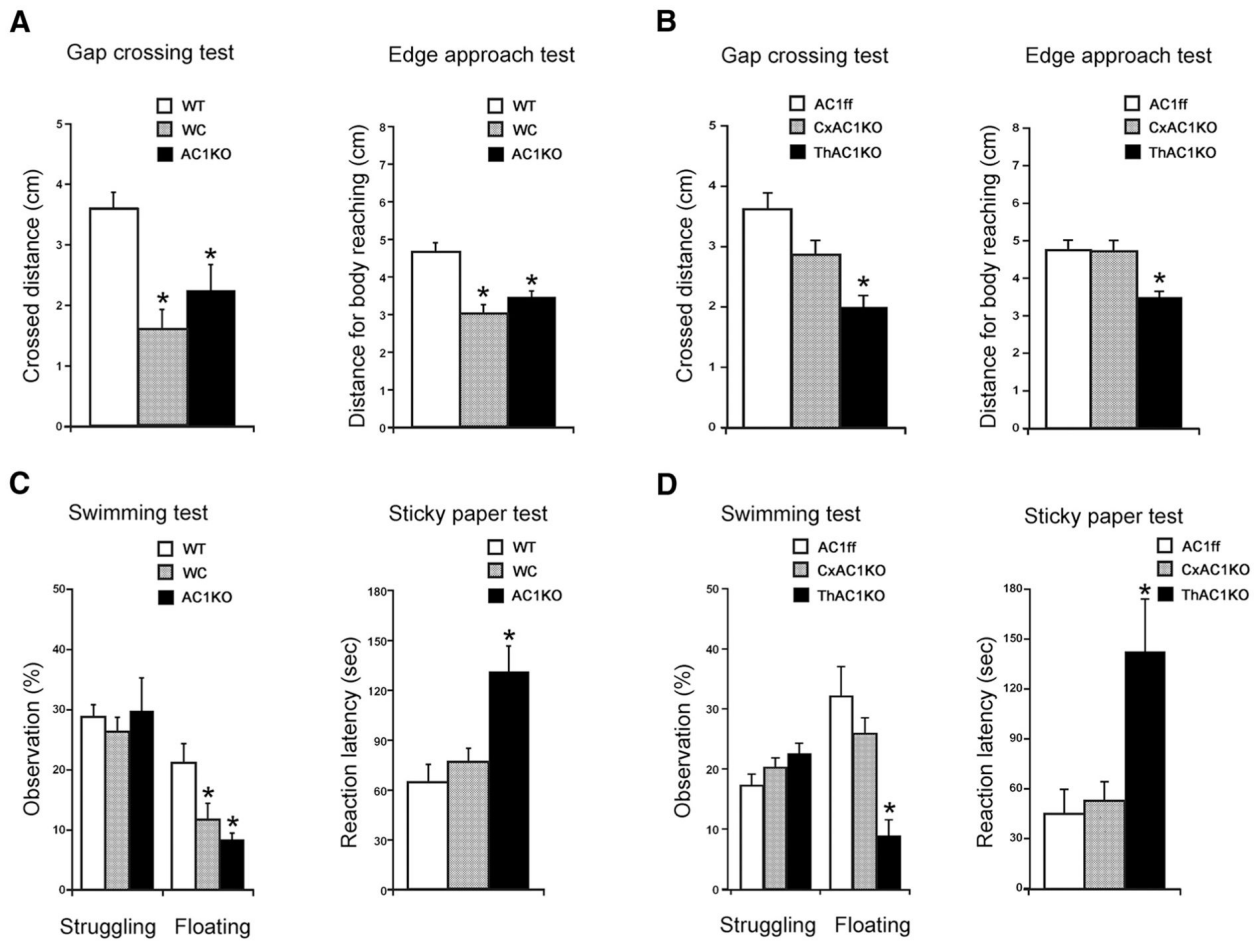
Sensorimotor behavior comparisons. ***A*, *B***, Gap crossing and edge approach tests. WT mice crossed longer gap distances than WC or AC1KO mice, *F*_(2,23)_ = 8.276, *p* = 0.0022. ThAC1KO mice crossed shorter gap distances than control AC1^flox/−^(AC1ff) mice, *F*_(2,21)_ = 12.70, *p* = 0.0002, while CxAC1KO mice performed similar to controls. In the edge approach test, WC and AC1KO mice were significantly impaired compared with WT controls, *F*_(2,23)_ = 14.071, *p* = 0.0001. Likewise, ThAC1KO mice reached significantly shorter distances compared with CxAC1KO or AC1ff controls, *F*_(2,21)_ = 10.971, *p* = 0.0005. ***C*, *D***, Swimming and paw sensation tests. In the swimming test, AC1KO and WC mice showed a similar level of struggling, *F*_(2,23)_ = 0.190, *p* = 0.8283, but significantly shorter periods of floating than WT controls, *F*_(2,23)_ = 6.572, *p* = 0.0055. Cx, ThAC1KO, and AC1ff controls all showed a similar level of struggling, *F*_(2,21)_ = 2.452, *p* = 0.1104, while ThAC1KO mice floated significantly shorter time than either CxAC1KO or AC1ff controls, *F*_(2,21)_ = 11.754, *p* = 0.0004. In the sticky paper test, AC1KO mice displayed higher latencies for licking off the paper stuck on their hind paw, *F*_(2,23)_ = 8.175, *p* = 0.0021. ThAC1KO mice also showed higher latencies licking the paper compared with either CxAC1KO or AC1ff mice, *F*_(2,21)_ = 4.636, *p* = 0.0215. All data are expressed as mean ± SEM * indicates significant differences between strains.

We conducted two whisker-dependent behavioral tests. In one, mice had to assess the distance of a gap with their whiskers and cross the gap under infrared lighting; in the other, the mice had to reach the edge of a desk by using whiskers when suspended by the tail. Mice made use of the tip of their nose, paws, or whiskers to gauge the distance to cross the gap in the dark. WT mice crossed longer gap distances compared with WC or AC1KO mice (Fig. 2A). ThAC1KO mice crossed shorter gap distances than either the CxAC1KO or their controls, AC1^flox/−^ mice, under infrared lighting condition (Fig. 2B). These data indicate that whisker clipping significantly impairs the performance of gap crossing and lack of AC1 in the thalamus, but not in the cortex.

The horizontal-approach test requires tactile sensing as the animal approaches a surface when suspended in the air. Whisker-clipped mice or AC1KO mice were significantly impaired in this test when compared with WT controls. (Fig. 2B) Similarly, ThAC1KO mice showed shorter distance of reach when compared with either CxAC1KO or AC1^flox/−^ mice.

While swimming, whiskers help keep balance (Carvell and Simons, 1990). WC mice and AC1KO mice showed a similar level of struggling, paddling with all limbs in the water, compared with WT mice. However, WC mice and AC1KO mice displayed a significant level of impairment in floating when compared with their WT controls (Fig. 2C). ThAC1KO mice also showed a decreased floating ratio, but similar level of struggling compared with either CxAC1KO or their controls (Fig. 2D). These data indicate that whiskers play a significant role in floating, and the lack of AC1 function, especially in the thalamus, results in impaired behavioral performance in water.

For whisker-independent tactile sensation, we used the sticky paper test. Global AC1 deletion, or thalamus-specific AC1 deletion, involves not only the whisker representation areas of the somatosensory system but the paw representations as well. This task requires mice to detect and remove a piece of paper stuck on their hind paw (Crawley, 1999; Komotar et al., 2007). WT and WC mice showed a similar latency for licking the paper off their hind paw. AC1KO mice, however, showed significantly delayed latencies in licking the piece of paper compared with the WT controls (Fig. 2C). ThAC1KO mice also showed significantly longer latencies than either CxAC1KO or the controls (Fig. 2D). These data indicate that lack of whiskers has no impact, but global or thalamus-specific loss of AC1 function has a significant impact on hind paw tactile sensation.

**Figure 3:**
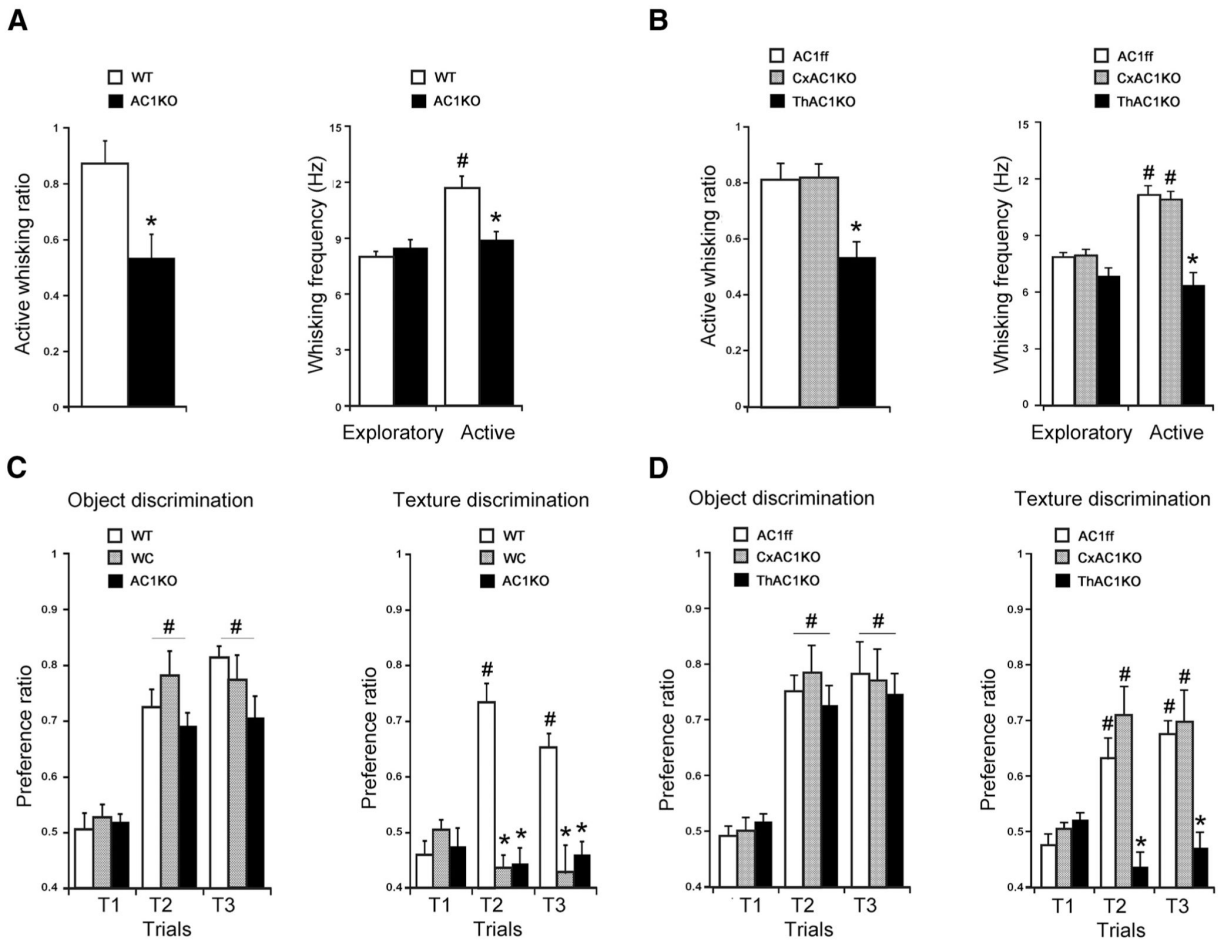
Whisking and object recognition tests. ***A***, Whisking behavior comparisons between AC1KO, WT, and WC mice. AC1KO mice showed a lower ratio of active whisking whisker contact with an object compared with WT mice; *t*_(16)_ = 3.4495, *p* = 0.0063. WT mice displayed a clear difference in vibration frequencies between symmetry (nontouch) and active (touch) whisking—the frequencies of active whisking were higher than symmetry whisking—while AC1KO mice showed similar frequencies between symmetry and active whisking. This was supported by a two-way ANOVA; strain: *F*_(1,14)_ = 7.007, *p* = 0.0191, whisking type: *F*_(1,14)_ = 25.373, *p* = 0.0002, and the interaction between strain and whisking type: *F*_(1,14)_ = 16.174, *p* = 0.0013. ***B***, Whisking comparisons between CxAC1KO, ThAC1KO, and control AC1^flox/−^ (AC1ff) mice. ThAC1KO mice showed a lower ratio of active whisking during object contact with whiskers than CxAC1KO or their controls (AC1ff); *F*_(2,21)_ = 9.223, *p* = 0.0013. CxAC1KO and AC1ff mice displayed a clear difference in vibration frequencies between symmetry (nontouch) and active (touch) whisking—the frequencies of active whisking were higher than symmetry whisking—while ThAC1KO mice show similar frequencies between symmetry and active whisking. This was supported by a two-way ANOVA; strain: *F*_(2,21)_ = 18.42, *p* = 0.0035, whisking type: *F*_(1,21)_ = 72.46, *p* = 0.002, and the interaction between strain and whisking type: *F*_(2,21)_ = 17.273, *p* = 0.0054. ***C***, Texture and object discrimination. In the object discrimination test, there were no significant strain differences throughout trials, while the preference ratio on trials 2 and 3 were higher than trial 1; a two-way ANOVA: strain: *F*_(2,23)_ = 1.416, *p* = 0.263; trial: *F*_(2,46)_ = 67.984, *p* = 0.0001; and strain × trial: *F*_(2,46)_ = 1.668, *p* = 0.1738. However, in the texture discrimination test, WT mice clearly showed a higher preference toward an unfamiliar textured object than familiar one on trials 2 and 3, while AC1KO and WC mice did not show significantly lower preference than WT control on trial 1, but did on trials 2 and 3; a two-way ANOVA: strain: *F*_(2,23)_ = 26.438, *p* < 0.0001; trial: *F*_(2,46)_ = 0.3997, *p* = 0.634; and strain × trial: *F*_(2,46)_ = 13.910, *p* < 0.0001. In the object-discrimination test, there were no significant strain differences between CxAC1KO, AC1ff, and ThAC1KO throughout trials, while the preference ratio on trials 2 and 3 were higher than on trial 1; a two-way ANOVA: strain: *F*_(2,21)_ = 0.24, n.s.; trial: *F*_(2,42)_ = 49.57, *p* = 0.0003; and strain × trial: *F*_(2,42)_ = 0.38, n.s. In the texture-discrimination test, CxAC1KO and AC1ff mice displayed a higher preference toward an unfamiliar textured object than familiar one on trials 2 and 3, while ThAC1KO mice showed similar preference throughout all three trials; a two-way ANOVA: strain: *F*_(2,21)_ = 12.45, *p* = 0.0087; trial: *F*_(2,42)_ = 13.96, *p* = 0.0075; and strain × trial: *F*_(2,42)_ = 9.59, *p* = 0.0082. All data are expressed as mean ± SEM. * indicates significant differences between strains, # indicates significant differences between trials.

How does AC1 loss of function affect whisking and object detection with whiskers? Mice are capable of whisking at high frequencies during encounters with objects while navigating in the dark (Jin et al., 2004). We observed the patterns of whisker movements with a high-speed video camera by recording from above the cage. We found two significantly different patterns of whisking: exploratory whisking, which consists of small symmetric vibrations of whiskers in a horizontal direction when not contacting an object, and active whisking, which consists of dynamic, bundled movement of whiskers forward when contacting an object (Brecht et al., 1997; Towal et al., 2011). WT mice displayed a clear difference in vibration frequencies between exploratory and active whisking; the frequencies of active whisking (11.72 ± 0.61 Hz) were higher than those of exploratory whisking (8.06 ± 0.61 Hz) (Fig. 3A). In contrast, AC1KO mice showed similar whisking frequencies between nonoriented and oriented exploration. The ratio of active whisking (in total observed whisking) was high for WT mice compared with that seen in AC1KO mice (Fig. 3A). There were similar asymmetries in the whisking pattern between the ThAC1KO, CxAC1KO, and AC1^flox/−^ mice. CxAC1KO and AC1^flox/−^ mice showed an increased frequency in active whisking compared with that seen during exploratory whisking. However, ThAC1KO mice displayed similar frequencies in both the exploratory and active whisking (Fig. 3B). ThAC1KO mice also displayed a significantly lower ratio of active whisking when contacting an object compared with either that of CxAC1KO or AC1^flox/−^ controls (Fig. 3B). These data indicate that normal mice typically whisk at higher frequencies during exploration and contacting an object, while mice impaired in AC1 function in the thalamus use oriented whisking during contact with an object at a lower rate.

We used a unique set of tasks requiring recognition of novel objects through visual and tactile cues. Both tasks are based on the innate curiosity of mice for novelty compared with the familiar. The first task was an object-shape discrimination test in which mice were expected to discriminate between two objects presented in their home cages under red lighting. When two identical objects were presented on trial 1, all strains of mice, including WT, WC, and AC1KO, showed exploratory whisking toward the two objects. When we changed one of the objects to an unfamiliar, differently shaped object in trials 2 and 3, mice in all three groups investigated the unfamiliar object for longer periods of time compared with the familiar object. A comparison between ThAC1KO, CxAC1KO, and AC1^flox/−^ mice revealed a similar pattern of object investigation through trials. All mice showed exploratory investigation (whisking, sniffing) toward two objects on trial 1, and longer periods of investigation toward an unfamiliar object over familiar object on trials 2 and 3 (Fig. 3C, D). These data indicate that mice, even when all their whiskers are clipped or lack AC1 function, are able to distinguish between and remember two differently shaped objects under the red-lighting condition.

In the object-texture discrimination test, the shape of the objects was identical but we changed the surface texture between trials. Mice were tested to distinguish the objects based on their texture differences in the dark. When the object’s texture was changed on trial 2 and 3, WT mice clearly showed a higher level of investigation toward an unfamiliar textured object compared with the familiar textured object (Fig. 3C). However, mice with clipped whiskers or those lacking AC1 function showed similar duration of investigation of the unfamiliar and familiar textures on trials 2 and 3 (Fig. 3C). CxAC1KO and AC1^flox/−^ mice revealed a similar pattern of investigation as WT mice (Fig. 3D); they showed similar investigation toward two identical objects on trial 1 and increased investigation toward an unfamiliar texture over the familiar texture on trials 2 and 3. In contrast, ThAC1KO mice showed similar levels of investigation of the two objects throughout the three trials.

**Figure 4:**
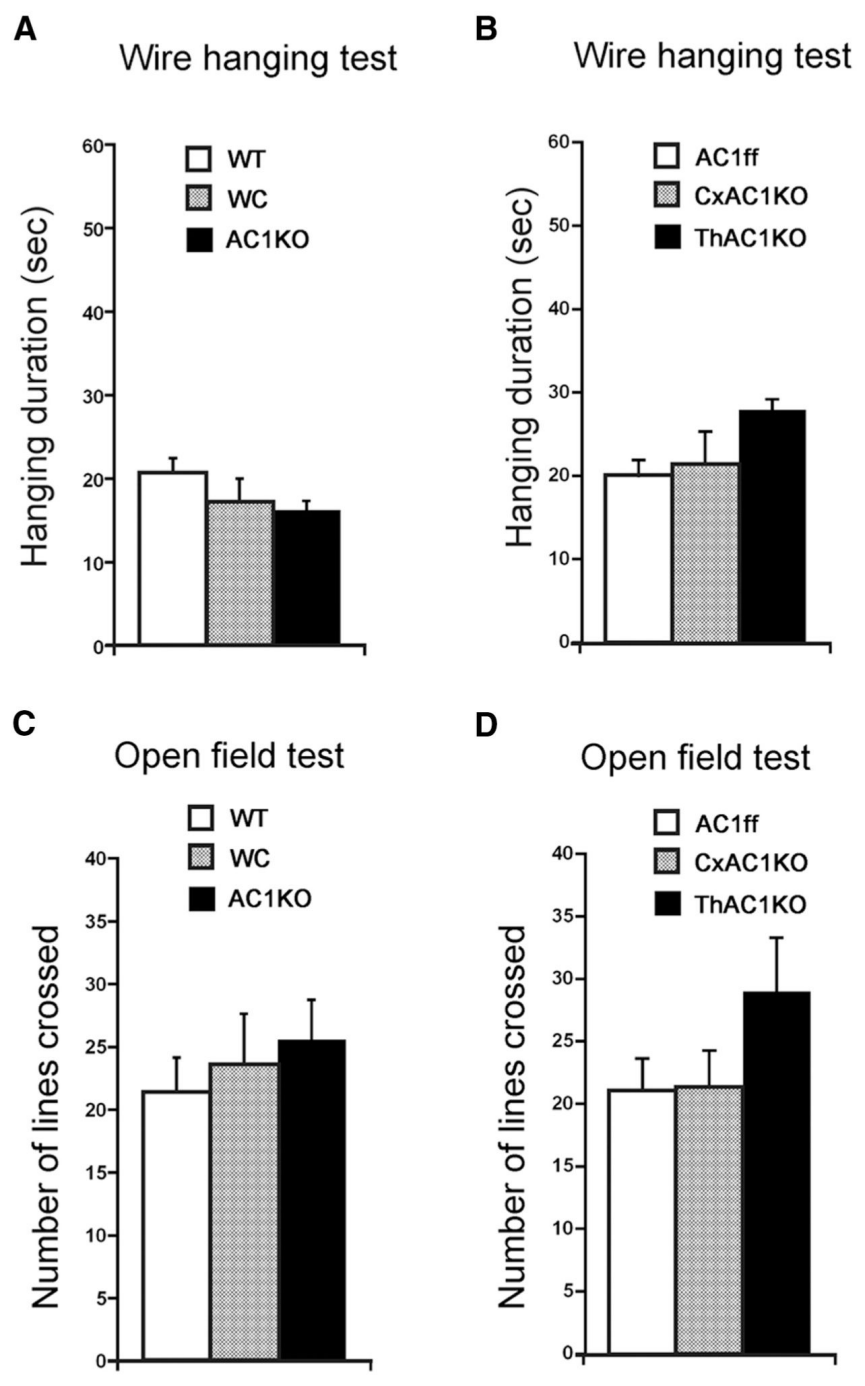
General motor behavior. ***A***, ***B***, In the wire-hanging test, there were no significant differences between any of the strains. ***C***, ***D***, In open-field exploration tests, there was no significant difference in locomotion, *F*_(2,23)_ = 0.317, *p* = 0.7316 and *F*_(2,21)_ = 1.821, *p* = 0.1865.

We did not find any significant differences in physical motor behaviors between any of the lines in our study. We used the wire-hanging test to evaluate motor function and physical performance (Crawley, 1999). We placed the mice on an inverted cage top and recorded the latencies of dropping to the cage floor in three trials. WT mice showed a latency for falling similar to WC and AC1KO mice (Fig. 4A). ThAC1KO mice also showed latencies similar to CxAC1KO and AC1^flox/−^ (Fig. 4B). We observed the behavior of mice in an open-field apparatus to assess their exploratory locomotor activity. There were no significant differences between the groups (WT, WC, and AC1KO or ThAC1KO, CxAC1KO, and AC1^flox/−^ mice; Fig. 4C, D).

**Figure 5:**
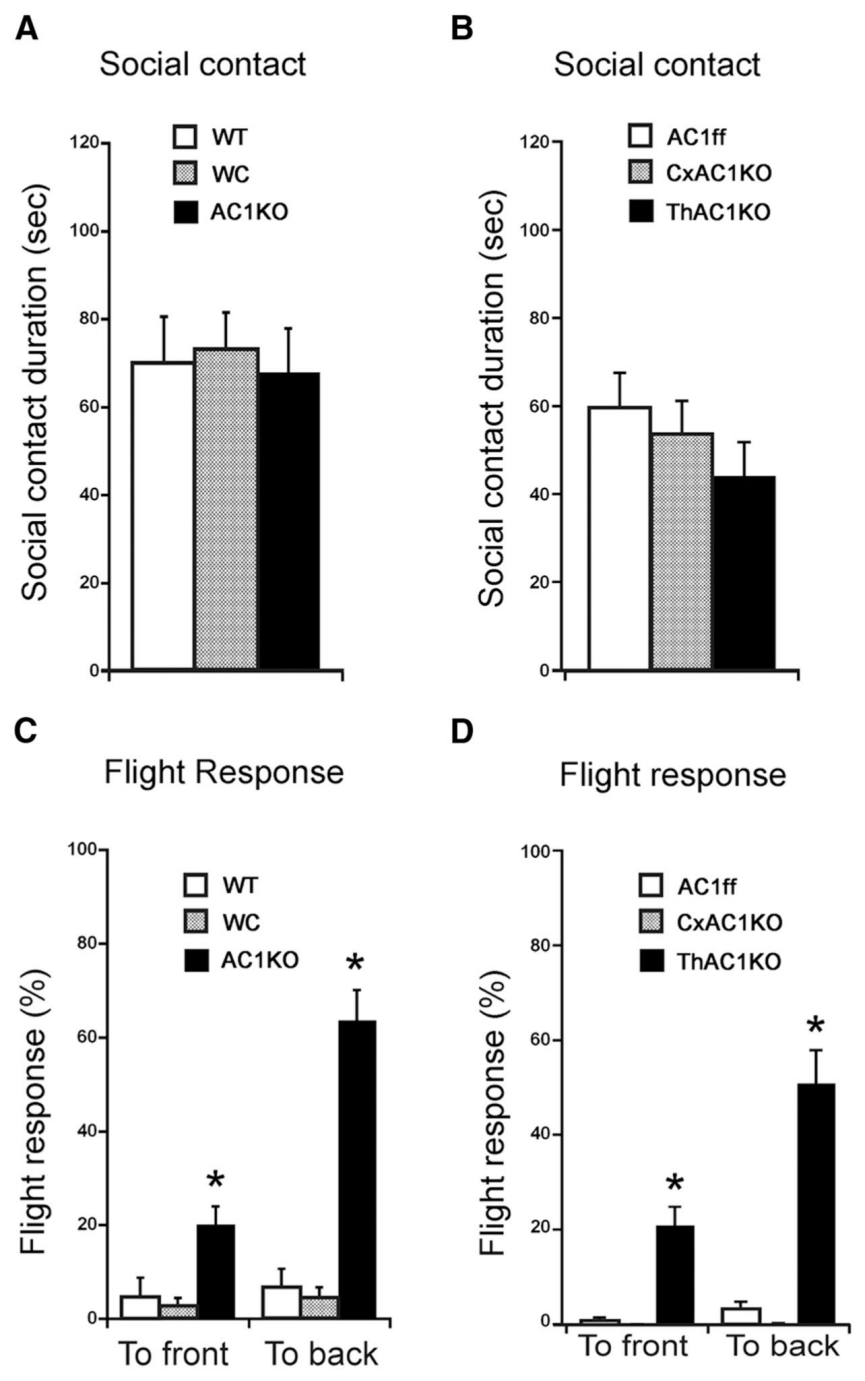
Social behavior. ***A***, ***B***, In the social behavior test, there was no difference in the duration of social contacts, *F*_(2,23)_ =0.084, *p* =0.9201. However, AC1KO mice displayed a notable increment of flight response to the intruder’s contacts from the front, *F*_(2,23)_ =7.662, *p* =0.0028, and back sides, *F*_(2,23)_ =48.434, *p* =0.0001, compared with WT or WC mice. ***C***, ***D***, In comparisons of the second group of mice, there was no difference in the duration of social contacts, *F*_(2,21)_ =5.896, *p* =0.093. However, ThAC1KO mice displayed a greater flight response to the intruder’s contacts from the front, *F*_(2,21)_ =25.792, *p* = 0.0001, and back sides, *F*_(2,21)_ = 46.706, *p* < 0.0001, compared with either CxAC1KO or AC1ff mice. All data are expressed as mean ± SEM * indicates significant differences between strains, # indicates significant differences between trials.

In the last behavioral experiment, we examined the social interaction among pairs of mice by the patterns of social contacts toward an intruder and flight responses to the frontal or rear approaches by the intruder. There was no difference in the amount of social contacts between AC1KO, WC, and WT mice (Fig. 5A). ThAC1KO mice showed an amount of social contacts similar to CxAC1KO and AC1^flox/−^ mice (Fig. 5B). Interestingly, AC1KO mice displayed a notable increment of flight response to the opponent’s contacts from both the front and the back compared with B6 mice with or without intact whiskers (Fig. 5C). ThAC1KO mice also showed a higher flight response to frontal and back approaches compared with CxAC1KO and AC1^flox/−^ mice (Fig. 5D). These findings suggest that global or thalamic, but not cortical, AC1 loss of function produces abnormal tactile sensation during social contacts, which results in pronounced flight response during social interaction.

## Discussion

Adenylate cyclases (ACs) are enzymes responsible for the synthesis of cAMP from ATP, and at least nine isoforms of ACs have been cloned and characterized in mammals (Hanoune and Defer, 2001). They are expressed in tissue-and cell-type-specific manners and their expression is developmentally regulated. In the brain, calcium/ calmodulin-activated AC1 and AC8 have drawn much attention through a wide spectrum of functions ranging from developmental refinement of neural circuits to olfaction, addiction, memory formation, pain, depression, and neurodegeneration (Hanoune and Defer, 2001; Wang and Storm, 2003; Wang and Zhang, 2012; Nicol and Gaspar, 2014).

There are several reports on the whisker-specific pattern deficits in the somatosensory system of mice with various types of AC1 loss of function. AC1 is expressed at all levels of the somatosensory pathway, from early developmental periods forward (Nicol et al., 2005). In the *barrelless* AC1KO mice, whisker-specific neural patterns are present in the brainstem (barrelettes) and there are only minor defects in thalamic barreloids (Welker et al., 1996; Abdel-Majid et al., 1998). However, in these animals, barrels are absent in the SI cortex and the thalamo-cortical axon terminal segregation is compromised (Welker et al., 1996; Gheorghita et al., 2006; Iwasato et al., 2008). Targeted gene deletion in excitatory neurons of the cortex does not reproduce the *barrelless* phenotype of the AC1KO mice but yields minor structural alterations (Iwasato et al., 2008). Interestingly, deletion of the AC1 in the sensory thalamus leads to a *barrelless* phenotype (Suzuki et al., 2013), suggesting that thalamic AC1 is the most critical player in the formation of cortical barrels.

A surprising finding in the *barrelless* AC1KO mice was that, despite the absence of barrels and TCA terminal segregation, whisker stimulation activated topographically appropriate areas in the face representation of the SI cortex, and morphologically, TCA terminal bouton density was concentrated in such topographically appropriate zones (Abdel-Majid et al., 1998; Gheorghita et al., 2006). In the present study, we confirmed this finding with activity-dependent immediate early gene expression and conducted behavioral testing to compare and contrast how thalamic, cortical, or global AC1 deletions, with specific roles in patterning of the somatosensory cortical body map, affect the sensorimotor repertoire of the animal. Our results show that thalamic AC1 gene impairment, similar to global AC1 knockout, yields the most pronounced behavioral deficit. Considering that both strains of mice lack, or have significantly impaired, patterning of the somatosensory body map in the neocortex, sensorimotor behavioral deficits seen in these animals indicate the importance of cortical pattern formation.

Mice are nocturnal and primarily use their whiskers to navigate in the dark and explore objects that they encounter. Whiskers are essential for estimating gap distances, depth of cliffs, spatial orientation, object localization, and texture discrimination, as well as in huddling and fighting behaviors (Hutson and Masterton, 1986; Carvell and Simons, 1995; Landers and Sullivan, 1999; Sarna et al., 2000; Krupa et al., 2001; Ahissar and Knutsen, 2008; O’Connor et al., 2010; Kleinfeld and Deschenes, 2011; Deschenes et al., 2012). We characterized and compared several behavioral outcomes of whisker clipping in normal mice with barrels (WC) and mice with all their whiskers intact but lacking barrels (AC1KO). Our results show that not only the whiskers but also the barrel patterning in the SI cortex is important in execution of a normal behavioral repertoire in a variety of sensorimotor tasks. Furthermore, in region-specific impairment of AC1 function in the brain, thalamic impairment appears to have far more drastic effects than that of cortical impairment. While we cannot rule out possible changes in the microcircuitry of the sensory thalamus, our results shed light on to the role of AC1 function in different levels of the somatosensory pathway and draw attention to the patterning of topographically aligned sensory maps in behavioral outcomes.

## Figures and Tables

**Table 1:** Experimental design: random-design

	Data structure	Type of test	Power
Fig. 2. gap distances, AC1KO, WC, and WT	Normal distribution	One–way ANOVA	1.0000
Fig. 2. gap distances, ThAC1KO, CxAC1KO, and control	Normal distribution	One–way ANOVA	1.0000
Fig. 2. edge approach, AC1KO, WC, and WT	Normal distribution	One–way ANOVA	1.0000
Fig. 2. edge approach, ThAC1KO, CxAC1KO, and control	Normal distribution	One–way ANOVA	1.0000
Fig. 2. swimming, struggling AC1KO, WC, and WT	Normal distribution	One–way ANOVA	0.8000
Fig. 2. swimming, floating AC1KO, WC, and WT	Normal distribution	One–way ANOVA	0.9999
Fig. 2. swimming, struggling ThAC1KO, CxAC1KO, and control	Normal distribution	One–way ANOVA	0.8507
Fig. 2. swimming, floating ThAC1KO, CxAC1KO, and control	Normal distribution	One–way ANOVA	1.0000
Fig. 2. sticky paper, AC1KO, WC, and WT	Normal distribution	One–way ANOVA	1.0000
Fig. 2. sticky paper, ThAC1KO, CxAC1KO, and control	Normal distribution	One–way ANOVA	0.9976
Fig. 3. active whisking frequency, AC1KO, WC, and WT	Normal distribution Normal distribution Normal distribution	Two–way ANOVA, strain Two–way ANOVA, type Two–way ANOVA, interaction	0.8831 1.0000 1.0000
Fig. 3. active whisking frequency, ThAC1KO, CxAC1KO, and control	Normal distribution Normal distribution Normal distribution	Two–way ANOVA, strain Two–way ANOVA, type Two–way ANOVA, interaction	1.0000 1.0000 1.0000
Fig. 3. object discrimination, AC1KO, WC, and WT	Normal distribution Normal distribution Normal distribution	Two–way ANOVA, strain Two–way ANOVA, trial Two–way ANOVA, interaction	1.0000 0.8100 1.0000
Fig. 3. object discrimination, ThAC1KO, CxAC1KO, and control	Normal distribution Normal distribution Normal distribution	Two–way ANOVA, strain Two–way ANOVA, trial Two–way ANOVA, interaction	0.9485 1.0000 0.9999
Fig. 3. texture discrimination, AC1KO, WC, and WT	Normal distribution Normal distribution Normal distribution	Two–way ANOVA, strain Two–way ANOVA, trial Two–way ANOVA, interaction	1.0000 1.0000 1.0000
Fig. 3. texture discrimination, ThAC1KO, CxAC1KO, and control	Normal distribution Normal distribution Normal distribution	Two–way ANOVA, strain Two–way ANOVA, trial Two–way ANOVA, interaction	0.8090 1.0000 0.8470
Fig. 4. open field locomotion, AC1KO, WC, and WT	Normal distribution	One–way ANOVA	0.8003
Fig. 4. open field locomotion, ThAC1KO, CxAC1KO, and control	Normal distribution	One–way ANOVA	0.9649
Fig. 5. social contacts AC1KO, WC, and WT	Normal distribution	One–way ANOVA	0.8008
Fig. 5. flight to front approach, AC1KO, WC, and WT	Normal distribution	One–way ANOVA	1.0000
Fig. 5. flight to back approach, ThAC1KO, CxAC1KO, and control	Normal distribution	One–way ANOVA	1.0000
Fig. 5. social contacts, ThAC1KO, CxAC1KO, and control	Normal distribution	One–way ANOVA	0.9999
Fig. 5. flight to front approach, ThAC1KO, CxAC1KO, and control	Normal distribution	One–way ANOVA	1.0000
Fig. 5. flight to back approach, ThAC1KO, CxAC1KO, and control	Normal distribution	One–way ANOVA	1.0000

## Significance Statement

The neuron-specific calcium-stimulated adenylate cyclase 1 (AC1) is important for refinement of topographic maps in the brain. AC1 is expressed at all levels of the somatosensory pathway and global or region-specific gene deletions lead to differential pattern phenotypes. Nothing is known about the behavioral consequences of such pattern defects related to loss of AC1 function. Here we show that global and thalamus-specific AC1 loss of function has more drastic behavioral impairments in somatosensorimotor and social behaviors than that with cortical loss of function. Our results shed light upon the role of AC1 function in different levels of the somatosensory pathway and the importance of neural patterning in topographic sensory maps in behavioral outcomes.
